# Logic Synthesis of Recombinase-Based Genetic Circuits

**DOI:** 10.1038/s41598-017-07386-3

**Published:** 2017-10-09

**Authors:** Tai-Yin Chiu, Jie-Hong R. Jiang

**Affiliations:** 1 Graduate Institute of Electronics Engineering, National Taiwan University, Taipei, 10617 Taiwan; 2 Department of Electrical Engineering, National Taiwan University, Taipei, 10617 Taiwan; 3 Genome and Systems Biology Degree Program, National Taiwan University, Taipei, 10617 Taiwan

## Abstract

A synthetic approach to biology is a promising technique for various applications. Recent advancements have demonstrated the feasibility of constructing synthetic two-input logic gates in *Escherichia coli* cells with long-term memory based on DNA inversion induced by recombinases. Moreover, recent evidences indicate that DNA inversion mediated by genome editing tools is possible. Powerful genome editing technologies, such as CRISPR-Cas9 systems, have great potential to be exploited to implement large-scale recombinase-based circuits. What remains unclear is how to construct arbitrary Boolean functions based on these emerging technologies. In this paper, we lay the theoretical foundation formalizing the connection between recombinase-based genetic circuits and Boolean functions. It enables systematic construction of any given Boolean function using recombinase-based logic gates. We further develop a methodology leveraging existing electronic design automation (EDA) tools to automate the synthesis of complex recombinase-based genetic circuits with respect to area and delay optimization. *In silico* experimental results demonstrate the applicability of our proposed methods as a useful tool for recombinase-based genetic circuit synthesis and optimization.

## Introduction

The development of synthetic biology shows the feasibility to implement computing devices with DNA genetic circuits in living cells. Synthetic cellular designs often intended to implement certain functions that make cells respond to specific environmental stimuli or even change their growth and cellular development. For instance, synthetic toggle switches^1^ and genetic oscillators^2–5^ can be used to control cell metabolism, synthetic counters^6^ can be potentially applied to the regulation of telomere length and cell aggregation, and genetic logic gates^7–10^ can achieve digital computation in response to stimulus input signals. In addition to these transcription-based DNA circuits, new emerging translational mRNA circuits^11^ are likely to have impact on mammalian regenerative medicine and gene therapy. Through the genetic engineering, synthetic cellular circuits are potentially useful to perform therapeutic and diagnostic functions.

For some situations where noxious chemical stimuli exist for many cell generations, the computational results from the synthetic circuits in parent cells are required to be propagated to their daughter cells so that the daughter cells can save time to respond to the environmental stimuli. To achieve this transgenerational memory, one possible method is to store the computational results in separate synthetic memory devices which can be duplicated in cell divisions. In the recent work of Siuti *et al*.^12^, a more efficient scheme for constructing synthetic cellular circuits with integrated logic and memory was proposed, where the computational result was automatically stored in the computing circuit configuration and the changes of configuration can be propagated to its descendant cells. The so-implemented circuits were built based on recombinases and tested in *Escherichia coli* cells and they showed a long-term memory for at least 90 cell generations. More recently, recombinase-based logic circuits has been applied in clinical uses. For instance, in recent work^13^ the authors demonstrate that biosensor made of recombinase-based logic gates can be used to detect pathological glycosuria in urine from diabetic patients. The ability to build complex recombinase-based logic circuits is an important step to enable widespread biomedical applications.

**Figure 1:**
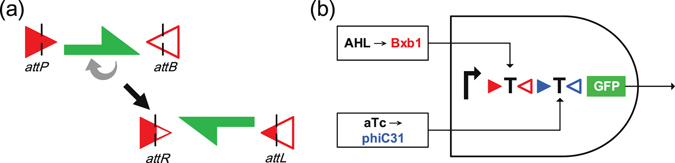
Recombinase-mediated DNA inversion and its application to the implementation of a logic gate. (**a**) Schematic illustration of the irreversible inversion of DNA sequences using serine recombinases. (**b**) Implementation of an AND gate using recombinases. The right-turn arrow represents a promoter; the red and blue triangles are the targeting sites of recombinases Bxb1 and phiC31, respectively; the letter T’s flanked by the targeting sites are transcription terminators; the green box represents the gene encoding the green fluorescent protein.

Specifically, the synthetic cellular circuits proposed by Siuti *et al*.^12^ used serine recombinases Bxb1 and phiC31 to implement various two-input logic gates. A serine recombinase targeting a pair of non-identical recognition sites known as *attB* (**att**achment site **b**acteria) and *attP* (**att**achment site **p**hage) is able to induce irreversible DNA inversion. As illustrated in Fig. 1(a), since the inversion makes the recognition sites become hybrid sites called *attR* and *attL* which cannot be targeted by the recombinase, no further inversion is allowed afterwards.

We illustrate how recombinases take part in the implementation of two-input logic gates with the two-input AND gate example shown in Fig. 1(b). (As a convention, in this paper we read a DNA sequence from left to right assuming the 5′-to-3′ direction of the coding strand). Let molecules AHL and aTc be the stimulus inputs to a cell and act as inducers activating the expressions of recombinases Bxb1 and phiC31, respectively. These recombinases when activated will irreversibly invert (flip) the DNA sequences flanked by their recognition sites (denoted by the colored triangle pairs). The DNA sequences being flanked can be a promoter, a transcription terminator, or a reporter, e.g., a green fluorescent protein (GFP). Inverting these DNA sequences will alter the output gene expression. In Fig. 1(b), two terminators were flanked by the recognition sites of recombinases Bxb1 and phiC31, and the output green fluorescent reporter is highly expressed only when both inducers AHL and aTc are in high concentration to activate BxB1 and phiC31 which together further flip and disable both terminators (denoted by letter “T”). Therefore, the circuit of Fig. 1(b) effectively implements a two-input AND gate. Note that such DNA sequence changes will survive through cell divisions and can be inherited to descendant cells in different generations. Hence the so-implemented logic function can achieve a long-term transgeneration memory.

Motivated by the viability and applicability of recombinase-based circuits, in this paper we formalize the construction of a general multi-input logic gate with its DNA sequence composed of series of promoters and transcription terminators targeted by multiple recombinases. We further characterize the set of Boolean functions realizable under such logic gates. In addition, we show a design flow for arbitrary Boolean function construction with cascaded recombinase-based logic gates. This automated design methodology is demonstrated by leveraging synthesis tool ABC^14^, an electronic design automation (EDA) tool developed at UC Berkeley, to synthesize cascaded multi-level recombinase-based circuits.

## Methods and Results

**Figure 2:**
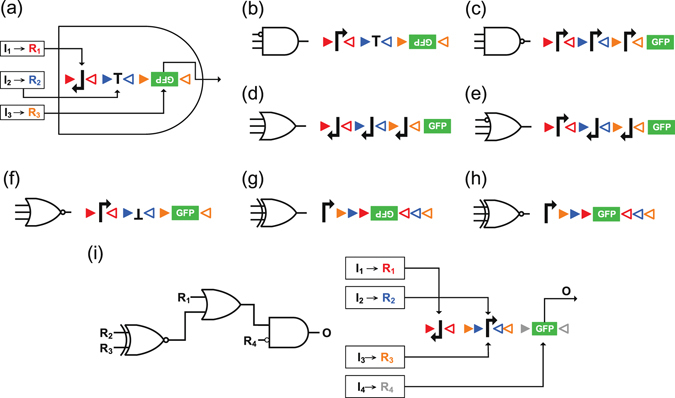
Examples of generalized multi-input recombinase-based logic gates. (**a**–**h**) Implementation of basic 3-input logic gates using recombinases. The inputs of each gate from top to down are recombinases *R*_1_, *R*_2_, and *R*_3_, respectively; inducer *I*_*i*_ monitored by the cell activates the expression of *R*_*i*_; the red, blue, and orange triangles denote the targeting sites of *R*_*i*_, *i* = 1, 2, 3, respectively. (**i**) Schematic illustration of a 4-bit non-basic logic function $O=({R}_{1}+\overline{{R}_{2}}\oplus {R}_{3})\overline{{R}_{4}}$ and corresponding implementation using recombinases.

To formalize the general multi-input gate construction, we use the three-input logic gates in Fig. 2(a–h) as examples to illustrate. Figure 2(a) shows a realization of a 3-input AND gate using three recombinases *R*_1_, *R*_2_, and *R*_3_, where molecule *I*_*i*_ is a stimulus input that activates the expression of recombinase *R*_*i*_, for *i* = 1, 2, 3. Then *R*_*i*_’s induce the inversions of their corresponding DNA sequence fragments. In order to express GFP in this gate, first we require *R*_1_ to invert the inverted promoter so that the RNA polymerase can bind to it and begin the transcription of the downstream DNA sequence in which the GFP gene resides. Second, *R*_2_ is needed to flip the terminator to avoid the termination of transcription before reaching the GFP gene. Third, *R*_3_ is demanded to upright the GFP gene for the RNA polymerase to initiate GFP production. Collectively, to have GFP highly expressed all *R*_*i*_’s must exist, and thus this circuit implements a 3-input AND gate. Note that this 3-input AND gate, where the promoter and the reporter gene GFP can be flipped by recombinases, is designed in a different fashion from the 2-input AND gate in Fig. 1(b), where only transcription terminators are inverted by recombinases. The additional choice of flipping the DNA fragments of promoter and GFP gives more flexibility for logic gate construction.

In Fig. 2(b–h) we present seven other basic 3-input gates implemented with recombinases. Special implementations with nested targeting sites are applied on the XOR gate in (g) and the XNOR gate in (h). In the XOR gate in (g), the existence of one or three recombinases results in one or three times of GFP gene flipping and thus making the upside-down gene become upright, while the existence of two recombinases makes the GFP gene flip twice and remain upside down. Similar situations happen in the XNOR gate in (h).

Since the implementations of multi-input gates are possible, we are not constrained to using only 3-input gates and basic gate types, such as AND, OR, NAND, NOR, XOR, and XNOR gates. Rather, we can construct complex logic gates with more inputs. Figure 2(i) shows an example of a 4-input logic circuit

$$O=({R}_{1}+\overline{{R}_{2}}\oplus {R}_{3})\overline{{R}_{4}},$$

which can be directly realized by a single 4-input complex logic gate, instead of cascading multiple two-input gates.

### Formalism of Recombinase-Based Logic Gates

#### Syntax of well-formed sequences

We define the following syntax to formalize the DNA sequences of logic gates constructed with recombinases. Here the basic elements composing a legal DNA sequence of a recombinase-based logic gate are “atomic terms”, including (inverted/non-inverted) transcription factors, (inverted/non-inverted) promoters, (inverted/non-inverted) genes, and targeting sites of recombinases. The syntax of DNA sequence forming a legal recombinase-based logic gate can be defined as follows.

**Definition 1** *An atomic term in a DNA sequence is a transcription terminator T*, *a promoter P*, *a gene G*, *an inverted transcription terminator* , *an inverted promoter* , *or an inverted gene* . *The syntax of an atomic term can be expressed in Backus-Naur Form as*
1


Let the targeting sites *attP* and *attB* of recombinase *r* in a DNA sequence be denoted as “{_*r*_” and “}_*r*_”, respectively. In the sequel, the subscripts of {_*r*_ and }_*r*_ may be omitted for brevity when they are clear from the context or immaterial to the discussion. Note that targeting sites “{” and “}” of a recombinase must appear in a pair.

**Definition 2** *The syntax of a* well-formed sequence (*wfs*) *is recursively defined as follows*.

2 $$\begin{array}{l}\langle wfs\rangle \,::=\,\langle atomic\,term\rangle \,|\,{\{\langle wfs\rangle \}}_{{r}_{i}}\,|\,\langle wfs\rangle \langle wfs\rangle .\end{array}$$

In this paper we concentrate on the special case of *one-gene wfs* (1g-wfs), where only one gene *G*, which is neither inverted nor sandwiched by targeting sites, appears at the end of the wfs and serves as the output. For example, , and  are 1g-wfs’s. Notice that under the 1g-wfs setting, the logic gate has a single output and the gene can only be transcribed in one direction from left to right.

A pair of targeting sites of a recombinase is called *basic* if it only flanks an atomic term. Otherwise, it is called *non-basic*. We call a 1g-wfs *basic* if it contains only basic pairs of targeting sites, and *non-basic* if it contains some non-basic pair of targeting sites. For example,  is a basic 1g-wfs. In contrast,  and  are non-basic 1g-wfs’s.

Furthermore, a non-basic pair of targeting sites can be nested. That is, a non-basic pair of targeting sites can be flanked by another pair of targeting sites. For instance,  has nested two pairs of targeting sites targeted by the recombinases *r*_3_ and *r*_4_.

We discuss the logic functions induced by basic and non-basic 1g-wfs’s in the following.

#### Semantics of well-formed sequences – Basic well-formed sequences

We first study some reduction rules of basic 1g-wfs’s. Let *σ* be the DNA sequence of a basic 1g-wfs excluding the output gene, that is, *σ* is a basic wfs without any gene. We denote a wfs without any gene as 0g-wfs. Because *σ* is made of components  ${\{T\}}_{{r}_{i}}$, and  for any component *C* in *σ* the sequence *σ* can be decomposed into

$$\sigma ={\sigma }_{1}C{\sigma }_{2},$$

where *σ*_1_ and *σ*_2_ are two 0g-wfs’s, if non-empty. We show that the logic gate induced by the 1g-wfs *σG* can be further reduced to an equivalent form according to the type of the component *C*.

When *C* is a transcription terminator *T*, then *σ* equals

3 $${\sigma }_{1}T{\sigma }_{2}G\equiv {\sigma }_{2}G.$$

This equivalence holds because any transcription that starts from *σ*_1_ to gene *G* is always blocked by the transcription terminator *T* in the middle, making *σ*_1_ *T* a don’t-care and thus removable.

When *C* is an inverted terminator , then *σ* equals4


This equivalence holds because the inverted terminator  never blocks the transcription and is thus removable.

When *C* is a promoter *P*, then *σ* equals

5 $${\sigma }_{1}P{\sigma }_{2}G\equiv P{\sigma }_{2}G.$$

This equivalence holds because no matter whether there is a transcription that starts from *σ*_1_ to *G* or not, a transcription can always start from the promoter *P*. Therefore, *σ*_1_ is a don’t-care and thus removable.

When *C* is an inverted promoter, then *σ* equals6


This equivalence holds because the transcription that begins at  proceeds across *σ*_1_ in the direction from right to left, it does not pass through *G*. As a result, the expression of *G* can not be initiated by  and thus  can be removed from the sequence.

When *C* is  since an atomic term *A* is equivalent to {*A*}_*r*_ for recombinase *r* being in low concentration (denoted *R* = 0 by treating *r* as a Boolean variable *R* of value 0) or  for recombinase *r* being in high concentration (denoted *R* = 1 by treating *r* as a Boolean variable *R* of value 1), the reduction rules for *C* can be easily extended from the previous rules as summarized below.

7 $${\sigma }_{1}{\{T\}}_{r}{\sigma }_{2}G\equiv \{\begin{array}{ll}{\sigma }_{2}G, & {\rm{for}}\,R=0\\ {\sigma }_{1}{\sigma }_{2}G, & {\rm{for}}\,R=1\end{array}$$

8

9 $${\sigma }_{1}{\{P\}}_{r}{\sigma }_{2}G\equiv \{\begin{array}{cc}P{\sigma }_{2}G, & {\rm{for}}\,R=0\\ {\sigma }_{1}{\sigma }_{2}G, & {\rm{for}}\,R=1\end{array}$$

**Figure 3:**
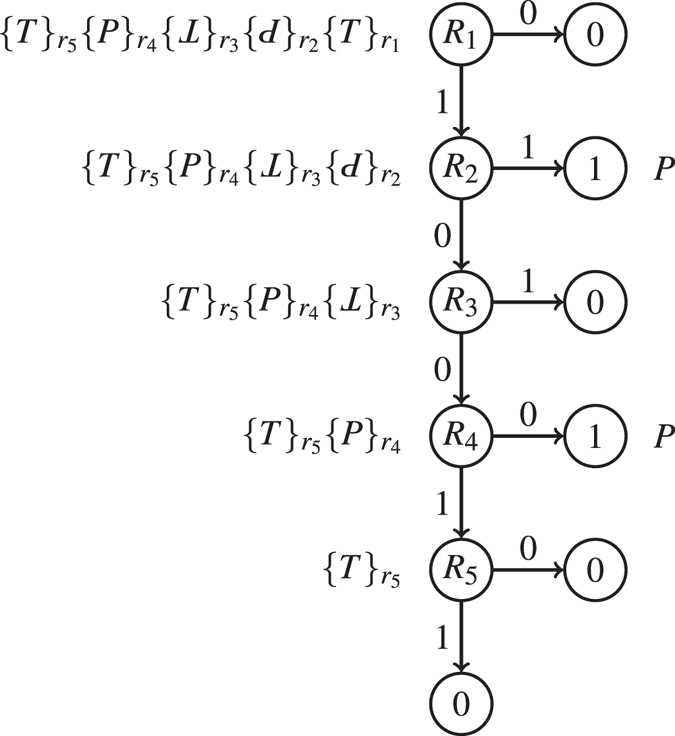
Decision list corresponding to 1g-wfs  Node labelled *R*_*i*_ is the decision for the logic value of *R*_*i*_. Nodes labelled 0 (resp. 1) stand for gene *G* cannot (resp. can) be expressed. The sequences beside nodes are the equivalent sequences after the corresponding (partial) decisions.

10


with the above analysis, we can derive the corresponding Boolean function of a given 1g-wfs. Consider the 1g-wfs *σG* with the sequence *σ* targeted by recombinases *r*_*i*_, $i=1,...,n$. Activating the expression of gene *G* requires the recombinases *r*_*i*_’s have adequate (high or low) concentrations so that the 1g-wfs *σG* effectively reduces to *PG*. The Boolean function induced by *σG* is determined through a series of decisions made by *r*_*i*_’s. In essence, it corresponds to a decision list^15^. To illustrate, consider the example  The decision list induced by the 1g-wfs *σG* is shown in Fig. 3. Note that given a sequence without non-basic targeting sites, the decisions always start from the rightmost to the leftmost components because a component closer to the gene may overwrite the effects imposed by the components on its left and thus it is of higher priority. Therefore, the Boolean function of *σG* is determined starting from *R*_1_ to *R*_5_. In order to reduce *σ* to *P* to express gene *G*, first we must require *R*_1_ to be 1. Otherwise if *R*_1_ = 0, *σ* becomes equivalent to a null sequence no matter what other *R*_*i*_’s are. Next, if we let *R*_2_ be 1, we can have an equivalent sequence equal to *P* as wished. Otherwise we can let *R*_2_ be 0 and look for other possibilities for the reduction to *P*. If *R*_2_ = 0, we can easily tell that the only possibility occurs when *R*_3_ and *R*_4_ are both 0 and that the logic of *R*_5_ never affects the reduction. Collectively, the logic function of the gate *σG* is derived as ${R}_{1}\cdot ({R}_{2}+\overline{{R}_{3}}\cdot \overline{{R}_{4}})$, where symbol “+” denotes Boolean disjunction, symbol “·” denotes Boolean conjunction, and symbol “−” or “!” denotes Boolean negation. In the sequel, we sometimes omit the conjunction symbol “·” in a Boolean expression.

In general, we can systematically convert any basic 1g-wfs to its corresponding logic function. To achieve this conversion, the operator Ω over a 1g-wfs is defined in Table 1. For an empty sequence ⊥, we define Ω[⊥] = 0. For example, the Boolean function of the 1g-wfs  is derived by

**Table 1:** Operators for parsing basic 1g-wfs *σCG*, with (non-empty) 0g-wfs *σ*, component *C*, and gene *G*, to logic function.

component *C*	operator Ω[*σC*]
*T*	0 · (Ω[*σ*])
*P*	1 + (Ω[*σ*])
{*T*}_*r*_	*R* · (Ω[*σ*])
{*P*}_*r*_	$\overline{R}+({\rm{\Omega }}[\sigma ])$
	1 · (Ω[*σ*])
	0 + (Ω[*σ*])
	$$\overline{R}\cdot ({\rm{\Omega }}[\sigma ])$$
	*R* + (Ω[*σ*])

#### Semantics of well-formed sequences – Non-basic well-formed sequences

We extend the above derivation of Boolean function to non-basic 1g-wfs’s by having the operator Ω over a 0g-wfs {*σ*}_*r*_ (which can be basic or non-basic) defined as11where  is the inverted sequence of *σ*. To understand equation (11), consider a 1g-wfs *σG* with only one pair of non-basic targeting sites. Suppose *σ* = {*σ*_1_}_*r*_, where *σ*_1_ is a basic 0g-wfs. Then *σ* is equal to *σ*_1_ when *R* = 0 and to , the inverted sequence of *σ*_1_, when *R* = 1. For example, the logic function for  can be obtained by


For a 1g-wfs with multiple (possibly nested) non-basic pairs of targeting sites, its logic function can also be directly derived by the Ω operator. For example, the logic function for  can be obtained by


Non-basic pairs of targeting sites can be exploited to efficiently construct special Boolean functions. One of such special functions is the parity function. An *n*-input odd parity function can be realized by the 1g-wfs


When there is an odd number of *R*_*i*_’s equal to 1, the 1g-wfs reduces to sequence *PG* and gene *G* can be expressed. Otherwise it reduces to sequence *G* and gene *G* cannot be expressed. On the other hand, the *n*-input even parity function can be realized by the 1g-wfs

$$\mathop{\underbrace{\{\cdots \{}}\limits_{n}P{\}}_{{r}_{1}}\cdots {\}}_{{r}_{n}}G.$$

### Construction of Multi-level Recombinase-Based Logic Circuits

**Figure 4:**
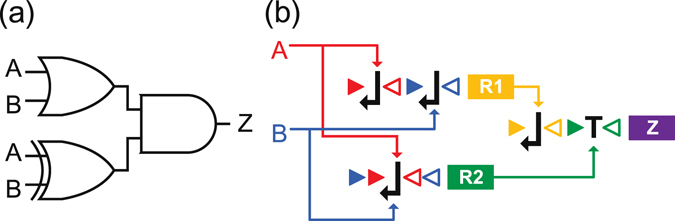
Example of a cascaded recombinase-based logic circuit. (**a**) Logic circuit of Boolean function *Z* = (*A* + *B*)(*A* ⊕ *B*). (**b**) The corresponding DNA implementation of the circuit in (**a**) with gate cascade. *A* and *B* denote the recombinase inputs of the overall circuit. The genes *R*1 and *R*2 encode the recombinases *r*_1_ and *r*_2_, respectively, which are the inputs to the downstream AND gate. The protein encoded by the gene *Z* is the output of the circuit.

With the recombinase-based logic gates built from 1g-wfs’s, we can cascade them to implement arbitrary complex multi-level circuits. For example, the logic function *Z* = (*A* + *B*)(*A* ⊕ *B*) can be implemented with the two-level circuit shown in Fig. 4(a), which is composed of an OR-gate, an XOR-gate, and an AND-gate. One possible DNA implementation of *Z* with cascade can be derived by converting each gate to their 1g-wfs realizations as shown in Fig. 4(b). The 1g-wfs’s that encode the genes *R*_1_, *R*_2_, and *Z* correspond to the OR, XOR and AND gates, respectively. The recombinases *r*_1_ and *r*_2_ as the inputs to the AND gate are the intermediate signals.

**Figure 5:**
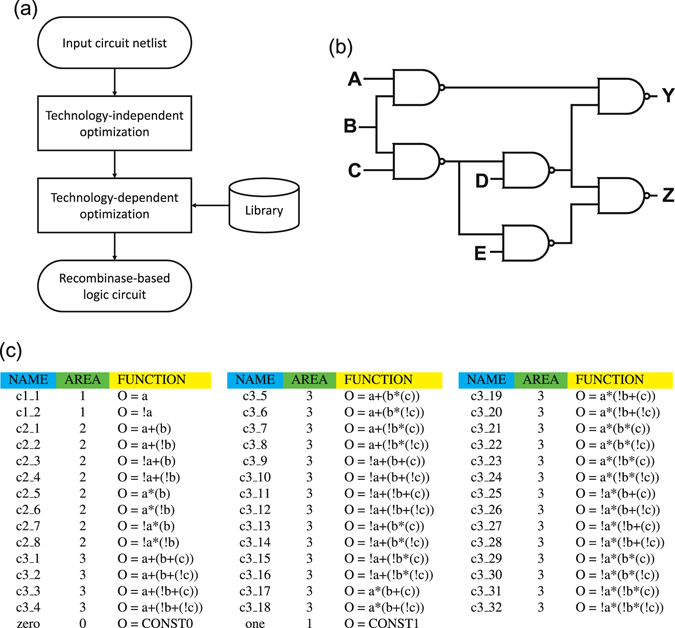
Illustration of the synthesis flow with an input circuit and a library of primitive gates. (**a**) Logic synthesis flow for the implementation of recombinase-based logic circuit. (**b**) Circuit diagram of an input circuit netlist example, ISCAS benchmark c17. Circuit c17 consists of six NAND gates with five inputs {*A*, *B*, *C*, *D*, *E*} and two outputs {*Y*, *Z*}. (**c**) Example of a library of DNA gates with area cost specified. The library contains 44 different cells and each cell corresponds to a DNA logic gate defined by a 1g-wfs with up to three inputs. The variables *a*, *b*, and *c* in a function specification represents the recombinase inputs to a gate, and the variable *O* denotes the gate output.

Because the basic 1g-wfs gates can implement decision list functions, they form a *functionally complete* set of primitive logic gates that can be composed to implement any Boolean function. Therefore the 1g-wfs gates can be collected as a library for the synthesis of complex logic circuits. By leveraging conventional logic synthesis tools in electronic design automation (EDA), recombinase-based logic circuits can be synthesized with the flow shown in Fig. 5(a). Given a Boolean function or circuit netlist as the input, it is first optimized by technology-independent techniques for circuit simplification. The simplified circuit is further optimized by technology-dependent techniques for technology mapping using the primitive gates in the given standard cell library. To achieve recombinase-based logic circuit synthesis, the main task is to provide the library while all other optimization tasks can be done using existing logic synthesis tools.

In this work, we adopt ABC^14^, an industrial-strength logic synthesis tool developed at UC Berkeley, for circuit synthesis and optimization. Given a circuit netlist, we first apply ABC to perform technology-independent optimization on the netlist, e.g., Boolean minimization to minimize the number of product terms and literals. We then use ABC to perform technology mapping to implement the area or performance optimized netlist using the 1g-wfs gates in the library.

To illustrate the synthesis flow, we consider implementing ISCAS benchmark circuit c17 shown in Fig. 5(b) with recombinase-based genetic circuit realization. The circuit consists of five inputs *A*, *B*, *C*, *D*, and *E*, and two outputs *Y* and *Z* with functions

12 $$\{\begin{array}{l}Y=AB+\overline{(BC)}D\\ Z=\overline{(BC)}D+\overline{(BC)}E.\end{array}$$

For area-driven synthesis of benchmark c17, there are 44 DNA gates defined by their 1g-wfs’s with up to three recombinase inputs. They are collected as the library as shown in Fig. 5(c). According to the experiment in the previous work^12^, where the promoters and transcription terminators used are roughly of the same length, we treat the area cost of both promoter and transcription terminator as unity. Therefore, the area cost of a DNA gate is defined as the number of atomic terms, excluding the output gene, that appear in the 1g-wfs of the gate. For example, the gate c3_1 corresponding to a 3-input OR gate has three inverted promoters as shown in Fig. 2(d). Hence, the area cost of c3_1 is counted as 3 units. By providing the c17 netlist and the library to ABC, the tool can perform optimization and technology mapping to find an area-optimized circuit composed of DNA gates of the library. Note that area minimization of a recombinase-based circuit effectively reduces the number of used promoters and terminators on the DNA strand implementation. Therefore, less effort is required to synthesize the intended DNA strand via DNA assembly methods, e.g., Gibson assembly^16^. More importantly, a shorter DNA sequence is more likely to succeed in vector insertion to deploy the genetic circuit into the host cell to conduct the intended computation.

**Figure 6:**
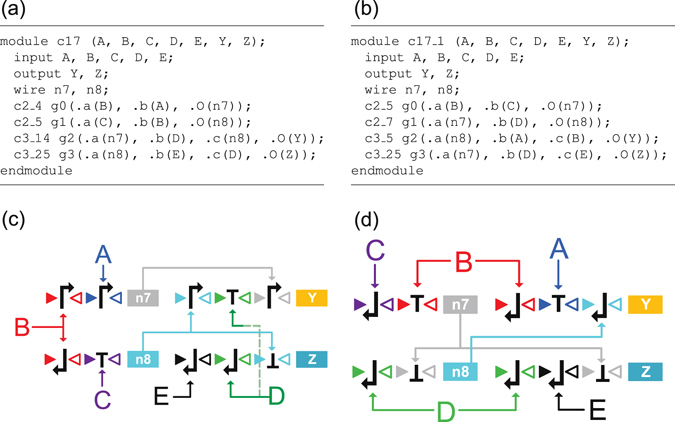
Synthesis results of circuit c17 in Verilog descriptions and in DNA circuit implementations. (**a**) Tool ABC synthesized c17 circuit in Verilog description. (**b**) Manually designed c17 circuit in Verilog description. (**c**) DNA circuit implementation of the ABC synthesized circuit in (**a**). (**d**) DNA circuit implementation of the manually designed circuit in (**b**). In both (**c**) and (**d**), symbols *A*, *B*, *C*, *D*, and *E* indicate the recombinase inputs, the proteins encoded by the genes *Y* and *Z* are the outputs of the circuit, and the DNA gates encoding recombinases *n*_7_ and *n*_8_ and proteins *Y* and *Z* are the gates g0, g1, g2, and g3, respectively, in the modules c17 and c17_1.

Figure 6(a) shows the result described in Verilog language of the synthesized c17 recombinase-based circuit using library gates listed in Fig. 5(c). The synthesized circuit comprises gates c2_4, c2_5, c3_14, and c3_25, and the total area cost is 10 units. Note that the naive DNA circuit implementation of c17 circuit by converting the digital logic gates in Fig. 5(b) to the corresponding DNA gates results in a total area cost of 12 units. Compared to the naive implementation, the area cost of the circuit synthesized by ABC technology mapping decreases. The logic functions of *Y* and *Z* in the synthesized circuit can be easily verified to be consistent with equation (12), implying the correctness of the synthesis result. The DNA circuit of module c17 in Fig. 6(a) is plotted in Fig. 6(c), where the symbols A, B, C, D, E, n7, and n8 represent some serine recombinases. In practice, to have recombinases achieve site-specific recombination in a synthetic genetic circuit, recombinases that have been reported to function outside their native hosts may be used. For example, well-reported recombinases^17–29^, such as *ϕ*C31, *ϕ*BT1, R4, BxB1, TP901-1, RV, SPBc, TG1, *ϕ*FC1, MR11, *ϕ*370, *ϕ*K38, A118, W *β*, and BL3 integrase, can be plausible molecular parts for realization of the recombinase signals in Fig. 6(c).

Note that there can be more than one area-optimized circuit of a logic function. For comparison, in Fig. 6(b) we show another manually designed DNA implementation of c17 circuit whose area cost is 10 units as well. The corresponding DNA circuit is plotted in Fig. 6(d). Notice that the two circuits in Fig. 6 differ not only in their constituent logic gates, but also in their logic depths. The circuit of Fig. 6(c) is of two logic levels, whereas that of Fig. 6(d) is of three logic levels. There are six longest paths in the former circuit:

$$\{\begin{array}{l}A\to n7\to Y,\\ B\to n7\to Y,\\ B\to n8\to Y,\\ B\to n8\to Z,\\ C\to n8\to Y,\\ C\to n8\to Z.\end{array}$$

They involve a cascade of two logic gates. On the other hand, there are two longest paths in the latter circuit:

$$\{\begin{array}{l}B\to n7\to n8\to Y,\\ C\to n7\to n8\to Y.\end{array}$$

They involve a cascade of three logic gates. In digital electronic circuits, a longer circuit path often corresponds to a longer propagation delay between circuit input and output signals. Similarly in biological circuits, a longer circuit path involves more transcription and translation cascades, resulting in a longer response time of output gene expression to input stimuli. Here, the former and latter circuits involve two (n7 and Y) and three (n7, n8, and Y) gene expression cascades, respectively. Therefore, although these two circuits have the same area cost, the circuit of Fig. 6(c) is preferred due to its better performance, i.e., shorter input-to-output response time. In addition, we will detail in Section Discussion that the delay optimization may present fewer foreign genes and thus impose less metabolic burden on the host cell. In the *in silico* experiments, we will synthesize circuits with area or performance optimized.

To demonstrate the feasibility of the proposed synthesis flow, we conduct *in silico* experiment on other 67 ISCAS benchmark circuits using recombinase-based DNA gates. We expanded the library such that it includes all 684 DNA gates with decision list functions up to five inputs. In the library, the area cost of a gate is determined by the number of atomic terms, excluding the output gene, appearing in its corresponding 1g-wfs. To reduce the number of gene expression cascades, we simply assume each logic gate is of the same unit delay. By specifying a unit delay for each gate in the library, the delay of a synthesized circuit equals the logic level, which equals the number of gene expression cascades in the longest path in the circuit. Consequently, under the unit delay model the performance-driven logic synthesis minimizes the delay time between input stimuli and output response in the synthesized recombinase-based circuit. Note that this simple unit delay model is not meant to reflect the timing behavior of actual biological systems, but to facilitate the logic synthesis algorithm to perform circuit logic level minimization.

The experimental results of 54 (out of the 67) circuits are shown in Table 2. The numbers of primary inputs/outputs, the number of inverters, and the number of logic gates (with the number of included buffers, if non-zero, reported in parentheses) are listed Columns 2, 3, and 4, respectively. The circuits were synthesized under two optimization settings: one for area optimization and the other for delay optimization. The results of area optimization are reported in Columns 5–7 and those of delay optimization are reported in Columns 8–10. For each synthesized circuit, its number of DNA gates, total area, and gate level are shown. In the naive implementations of benchmark circuits by simply converting the digital logic gates to the corresponding DNA gates, the total area of a DNA circuit can be roughly calculated as “#inverter” + 2 × “#gate”. Compared to the naive implementation, the circuits synthesized by ABC have much less area cost. Taking circuit b18 for example, we observe that the total area of the naive implementation is about 202110 which is much larger compared to the area 101870 of the area-optimized implementation and 105328 of the delay-optimized implementation. On the other hand, comparing area and delay optimized b18 circuits, delay optimization reduces the number of gate levels from 137 to 51 at cost of increasing area by 3500 units.

**Table 2:** Results of technology mapping of ISCAS benchmark circuits.

circuit name	benchmark profile			area optimization			delay optimization		
	#PI/#PO	#inverter	#gate (#buffer)	#DNA gate	area	#level	#DNA gate	area	#level
b03	34/34	16	106	91	217	7	79	228	4
b04	77/74	105	547	373	852	22	358	881	8
b06	11/15	7	32	25	56	6	24	62	3
b07	50/57	61	322	257	583	23	235	615	8
b08	30/25	26	123	90	224	12	85	233	5
b09	29/29	24	116	106	228	10	96	240	5
b10	28/23	32	140	100	260	11	96	298	4
b11	38/37	148	578	333	788	25	301	829	8
b12	126/127	113	831	707	1648	15	673	1786	6
b13	63/63	52	237	172	381	12	153	401	4
b14	277/299	1531	8236	2851	6947	124	2791	7749	18
b17	1452/1512	4474	26303	15344	37726	104	14802	39178	28
b18	3357/3343	20372	90869	43018	101870	137	40277	105328	51
b20	522/512	3068	16614	6119	14497	128	6111	16545	21
b21	522/512	3089	16938	6173	14724	121	6147	16631	21
b22	767/757	4491	24671	9302	22107	124	9286	24908	21
c432	36/7	40	120	79	200	25	91	276	11
c499	41/32	40	162	407	794	21	335	833	11
c880	60/26	63	320 (26)	234	530	26	208	553	8
c1355	41/32	40	506 (32)	394	781	19	328	878	10
c1908	33/25	277	603 (162)	336	690	28	271	736	13
c2670	233/140	321	872 (196)	409	956	19	400	1002	9
c3540	50/22	490	1179 (223)	566	1473	36	553	1649	14
c5315	178/123	581	1726 (313)	942	2202	25	908	2333	12
c6288	32/32	32	2384	1825	3709	89	1502	3995	38
c7552	207/108	876	2636 (534)	1149	2496	59	1084	2754	11
s208	19/10	35	61	39	100	8	39	105	3
s298	17/20	44	75	55	125	7	52	138	3
s344	24/26	59	101	82	178	11	67	175	4
s349	24/26	57	104	84	179	11	67	175	4
s382	24/27	59	99	78	172	8	70	191	3
s386	13/13	41	118	71	186	7	61	195	3
s400	24/27	56	106	80	173	9	76	220	3
s420	35/18	74	122	79	202	11	72	196	4
s444	24/27	62	119	75	169	9	74	210	3
s510	25/13	32	179	116	311	8	102	324	4
s526	24/27	52	141	88	202	11	79	223	3
s641	54/43	272	107	94	217	17	82	232	6
s713	54/42	254	139	90	212	16	85	237	6
s820	23/24	33	256	130	353	8	129	394	4
s832	23/24	25	262	132	358	9	135	406	4
s838	67/34	149	241	163	415	12	142	398	5
s1196	32/32	141	388	243	647	17	236	734	6
s1238	32/32	80	428	278	734	17	259	790	7
s1423	91/79	167	490	341	775	50	313	815	13
s1488	14/25	103	550	299	820	12	272	910	4
s1494	14/25	89	558	303	829	11	279	920	4
s5378	214/228	1775	1004	844	1843	14	780	1849	7
s9234	247/250	3570	2027	1065	2379	20	986	2442	9
s13207	700/790	5378	2573	2006	4075	26	1818	4153	9
s15850	611/684	6324	3448	2224	4946	35	2131	5018	16
s35932	1763/2048	3861	12204	6776	14953	9	5565	14718	5
s38417	1664/1742	13470	8709	6147	14319	23	5858	14551	10
s38584	1464/1730	7805	11448	7066	16905	37	6243	16433	11
**avg ratio**				**1.00**	**1.00**	**1.00**	**0.92**	**1.07**	**0.38**

## Discussion

### Area vs. Delay Optimization

To pursue area or delay optimization in genetic circuit synthesis is a matter of tradeoff, and may depend on the intended application and/or biological feasibility. Nevertheless, Table 2 reveals that when the library of recombinase-based logic gates is used in ABC for logic synthesis, delay optimization often achieves effective reduction (62% on average) in logic level, or circuit depth, with a slight increase (7% on average) in circuit area compared to area optimization. Taking the largest circuit b18 benchmark for example, from area to delay optimization, the area cost increases by 3.39% while the logic level decreases by 62.77%. Particularly, in practice since we are limited by the biotechnology and the metabolic burden, circuits to be synthesized cannot be as large as b18 benchmark, which only serves as a proof of concept. Instead, small circuits, such as b06, are more likely to be implemented. For benchmark b06, the area cost increases by 10.71% (56 to 62) and the logic level decreases by 50% (6 to 3) from area to delay optimization. Moreover, the delay optimization helps reduce metabolic burden (to be discussed below). These facts imply that delay-driven optimization may often be a proper objective for logic synthesis of recombinase-based genetic circuits.

### Metabolic Burden

One of the advantages of recombinase-based genetic circuits is its low metabolic burden imposed on the host cell^30^. Unlike a classic genetic circuit requiring continuous production of and action by activators or repressors to maintain the output gene expression, the output gene expression in a recombinase-based genetic circuit is determined by its DNA configuration, which is changed by DNA inversion or excision by recombinases; no further continuous recombinase supply and action is needed afterwards. This permanent configuration change is understood as a long-term (nonvolatile) memory, leading to the advantage of a lower metabolic burden on the host cell. This advantage may allow more complex genetic circuit implementation using recombinases. For example, recombinase-based finite state machines have been implemented in *E. coli* cells^31^. Moreover, a 6-input AND gate, a 2-data-input 4-select-input Boolean logic look-up table, a full adder, a full subtractor, and a half adder-subtractor were implemented in human embryonic kidney and Jurkat T cells^32^. Furthermore, we have shown recombinase-based logic gates can be adopted in the conventional logic synthesis flow for efficient circuit optimization. Because an efficient design can reduce metabolic burden and outperform an inferior counterpart even with the same functionality^33^, complex circuit implementation may benefit from the automation and optimization method proposed in this report.

Even with recombinase based construction, implementing a large circuit in a living cell may still be challenging due to the increase of metabolic burden^34^ caused by two major effects. First, a larger synthetic circuit requires more cellular energy to maintain its presence in the host cell^35^. Second, a large number of introduced genes will compete for the transcriptional and translational resources, resulting in resource redistribution^36^ and unexpected coupling among seemingly unconnected modules^37^, and thus leading to cell growth defects and poorly predictable circuit behavior. One approach to address these issues is to separate the target circuit into sub-circuits and implement the circuit across a consortium of host cells^7,38–40^. In particular, the consortium is divided into colonies of the same number of the sub-circuits. Each colony is composed of a strain implementing one of the sub-circuits. The sub-circuits are connected through cell-cell communication by wiring molecules (for example, quorum-sensing molecules and yeast pheromones) or metabolites like benzoic acid. Collectively, the whole cell population implements the target circuit. This distributed strategy may also apply to a large recombinase-based circuit implementation. For instance, the c17 circuit in Fig. 6(a) may be implemented by distributing the gates g0, g1, g2, and g3 into four strains of cells.

We note from Table 2 that when using recombinase-based logic gates as the library for a target circuit synthesis, the option of delay optimization introduces fewer DNA gates, each of which contains a gene, than the option of area optimization. Hence, delay optimization is preferred over area optimization due to a lower metabolic burden imposed by fewer foreign genes in the delay-optimized circuit.

### Experimental Steps for Circuit Realization

Given a target Boolean function to be implemented as a genetic circuit, our method can be applied as the first step to build the blueprint for the wet-lab construction by using the logic synthesis tool ABC to derive the area or delay-optimized circuit. The next task is to associate the abstract signals of the synthesized netlist with concrete biochemical parts, including promoters, recombinases, and genes, for wet-lab implementation. After this association step, the DNA molecule of the genetic circuit is readily to be constructed by Gibson assembly^16^, Unique Nucleotide Sequence (UNS) Guided assembly^41^, or other assembly methods. Note that the promoters used here should have the ability to strongly promote transcription. After the assembly, the DNA constructs are transformed/transfected into cells using a standard protocol, such as the polyethylenimine (PEI) protocol. The cells should be kept and maintained in custom or standard media, such as Luria-Bertani (LB) medium and Dulbecco’s Modified Eagle’s medium (DMEM), and grown for one to two days in a stimuli-free medium. To test the synthetic circuit, cells have to be exposed to stimuli and grown for several hours, and then the fluorescence response from cells is measured by a flow cytometer. For each sample of the measurement, the same number of cells should be used for consistency. After creating a gate using forward scatter (FSC) and side scatter (SSC) and applying a proper fluorescence threshold on each fluorescent protein channel, the percentage of cells in an ON state is determined by flow cytometry analysis.

### Alternative Genetic Circuit Construction with CRISPR/Cas9 Systems

Cas9 nucleases^42^ may possibly be exploited to achieve gene expression effects equivalent to what recombinases can achieve. For example, Cas9 nucleases are able to induce DNA deletion^43,44^, defective Cas9 nucleases (dCas9) can repress transcription by blocking transcriptional initiation or elongation^45^, and dCas9 fused with a transcriptional activator is capable of activating gene expression^46^. Specifically, DNA deletion of *P* and *T* may achieve an effect equivalent to inverting *P* and *T*, respectivley; transcription repression may achieve an effect equivalent to inverting *P* and ; transcription activation may achieve an effect equivalent to inverting  and *T*. These mechanisms allow CRISPR/Cas9 systems to be utilized as recombinase replacements for the implementation of decision list logic functions.

## Conclusion

In this paper, we generalized the two-input recombinase-based DNA logic gates to multi-input cases. We formalized the syntax of recombinase-based logic gate construction, and obtained the Boolean function semantics of well-defined DNA sequences of recombinase-based logic gates. We also showed how to synthesize multi-level recombinase-based logic circuits using existing logic synthesis tools. *In silico* experimental results demonstrate the feasibility and efficiency of our proposed methods as a tool for recombinase-based genetic circuit minimization. As recombinase-based logic circuits have been used in clinical biomarker detection and tested in human cells, our tool can be useful to automate complex recombinase-based circuit construction for biologists to implement advanced biomedical applications.
